# Joint angle estimation with wavelet neural networks

**DOI:** 10.1038/s41598-021-89580-y

**Published:** 2021-05-13

**Authors:** Saaveethya Sivakumar, Alpha Agape Gopalai, King Hann Lim, Darwin Gouwanda, Sunita Chauhan

**Affiliations:** 1 School of Engineering, Monash University Malaysia, Bandar Sunway, Malaysia; 2 Faculty of Engineering and Science, Curtin University Malaysia, Miri, Malaysia; 3 Department of Mechanical and Aerospace Engineering, Monash University Australia, Clayton, Australia

**Keywords:** Machine learning, Biomedical engineering

## Abstract

This paper presents a wavelet neural network (WNN) based method to reduce reliance on wearable kinematic sensors in gait analysis. Wearable kinematic sensors hinder real-time outdoor gait monitoring applications due to drawbacks caused by multiple sensor placements and sensor offset errors. The proposed WNN method uses vertical Ground Reaction Forces (vGRFs) measured from foot kinetic sensors as inputs to estimate ankle, knee, and hip joint angles. Salient vGRF inputs are extracted from primary gait event intervals. These selected gait inputs facilitate future integration with smart insoles for real-time outdoor gait studies. The proposed concept potentially reduces the number of body-mounted kinematics sensors used in gait analysis applications, hence leading to a simplified sensor placement and control circuitry without deteriorating the overall performance.

## Introduction

Qualitative human gait analysis is performed by evaluating changes of joint kinematics with respect to kinetics that has acted externally on the foot. Gold standard joint kinematics data acquisition is conducted using laboratory-based optical motion capture systems, which are equipped with multiple (at least six) tracking cameras. These cameras capture motion based on the movements of multiple reflective markers which are attached to segments and joints of the lower limb. However, laboratory-based gait analysis, although accurate, is not feasible for monitoring gait during acts of daily living (long-term) due to limited camera volume, movement restrictions caused by multiple marker attachments, and constraints related to calibrations.

Long-term gait monitoring is a vital element in the identification of gait abnormalities and also useful when tracking the progress of rehabilitation routines. With the emergence of compact and seamless wearable technology, long-term gait monitoring (and subsequently its analysis) has become a reality^1^. However, attaching multiple kinematic sensors for an extended duration causes inconvenience to the wearer, hence impacting natural gait. Furthermore, the initial calibration process, which requires the relation between sensors and anatomical segments before every use is challenging^2,3^. Although many supplementary techniques have been presented in the literature, the errors caused due to magnetic disturbances, drift, and noise of wearable kinematics sensors are not fully addressed^4^. In efforts to minimize the number of kinematics sensors and associated drawbacks, attention has turned to potential machine learning (ML) based gait parameter estimation techniques^3^.

ML-based gait estimations widely use artificial neural networks (ANNs)^5^ that are proven to be flexible and versatile for gait estimations. ANNs can learn meaningful gait relationships/characteristics using a non-linear approach and have proven to be a powerful method for diagnosis and treatment of human gait analysis^6^. Among the available ANNs, static neural networks are widely used in gait estimations^7–10^. Static neural networks are powerful estimators and have been applied in many applications including system modelling, signal processing, and control engineering^11–14^. A promising class of static neural networks is the wavelet neural network (WNN). WNNs are less prone to local minimum convergence and require the least number of hidden layer nodes compared to other conventionally used ANNs such as multilayer perceptrons (MLPs)^15^, thus reducing training time drastically^16^. WNN is an advanced ANN model, which combines static feedforward neural networks with wavelet functions for its hidden layer activation. The hidden layer nodes are activated using the wavelet analysis concept, in which a time-frequency decomposition is conducted to compress input data structures to fall within the bounds of the activation function. As such, WNNs are proven to hold higher compression abilities than MLPs. Activation functions with high compression abilities are beneficial when learning non-linear data characteristics that are often seen in gait data. Therefore, this paper presents a method that uses WNNs to estimate lower body joint angles as an attempt to reduce the reliance on kinematics sensors. Most studies in gait estimations have relied on kinematics sensors, which are inherently prone to placement errors, skin artefacts, and drift. An alternative is put forward in this paper, where we hypothesize that the load distribution can be used to estimate joint kinematic measurement via a WNN model.

The proposed WNN approach takes inputs only from vertical Ground Reaction Forces (vGRFs) to estimate lower body joint angles. Current methods inserted the full-length signal to the ANN, which include all the important as well as unimportant data points^17–25^. It is vital to consider feature selection methods that reduce the size of the input vector by selecting only important points of the signal to reduce training duration and complexity of network architecture^10^. We present the “Gait Intervals (GI)” method, which is a unique feature selection approach that selects data points based on the timings of the selected gait event intervals. The selected descriptive features provide overall characteristics of the signal in a short and crisp manner. The following contributions are presented:

1. Our method removed the complete reliance on kinematics sensors for gait applications and works as a solution to improve user experience and wearability. With no sensor attachments on joints and segments, the subjects are free to move their lower limb, thus not causing constraints on their natural walking style.
2. As a result of eliminating the reliance on kinematics sensors, this method further removes the requirement of using additional algorithms for error (e.g. skin artefacts) reduction associated with wearable kinematics sensors.
3. Our method acts as a generalized solution that does not require any subject-specific anthropometric information such as weight, height, and segment lengths.
4. The proposed algorithm estimates joint angles using prominent vGRF inputs corresponding to important foot contact forces. As a result, this study contributed to identifying the important positions of foot contact during gait, which could be of use in the future, for algorithms that are associated with kinetic insole sensors.

The paper is structured as follows. The next section describes, in brief, the “Background concept” section, followed by “Results and discussion” and “Conclusion” sections. A detailed explanation of the proposed methodology is presented in the “Methods” section stated at the end of this paper.

**Figure 1:**
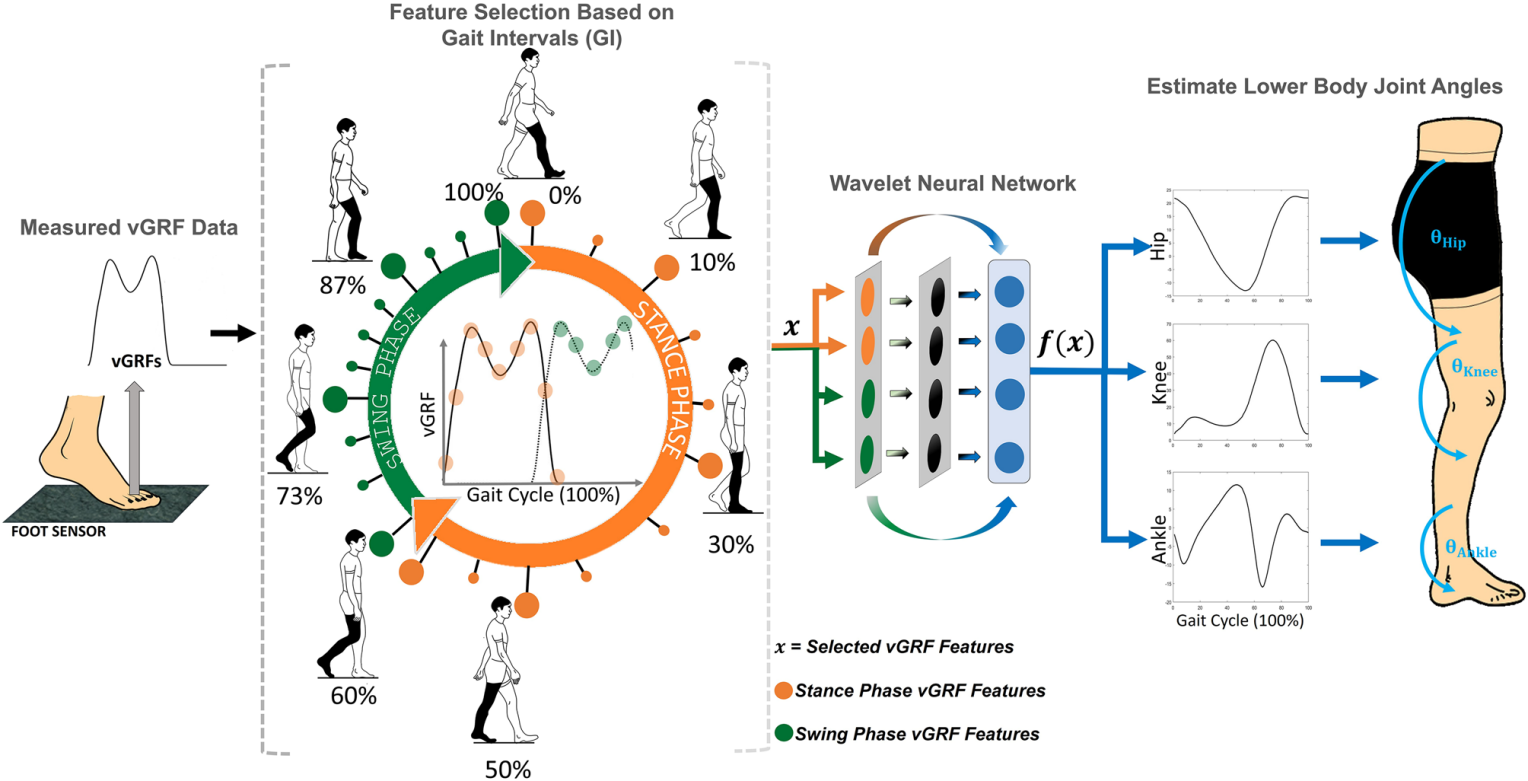
Overview of the proposed joint angle estimation concept. 22 vGRF features were selected using method GI. The features were selected based on gait intervals of nine primary gait events (denoted in bigger circles) and 13 intermediate gait events (denoted in smaller circles). Stance and swing phase features are distinguished in orange and green circles respectively. vGRF features were utilized as inputs to estimate ankle, knee, and hip joint angles using a WNN model.

## Background concept

An overview of the proposed concept is depicted in Fig. 1. vGRFs were used as inputs ($f$) to estimate lower body ankle ($\theta ^{a}$), knee ($\theta ^{k}$), and hip ($\theta ^{h}$) joint angles. A two-layered WNN was utilized as the medium to perform the estimations. The GI method is used to identify the network inputs by firstly extracting vGRF features based on the timing of the nine primary gait events. The nine gait events are; Initial Contact (IC), Opposite Toe Off (OTO), Heel Rise (HR), Opposite Initial Contact (OIC), Initial Toe Off (ITO), Next Toe Off (NTO), Feet Adjustment (FA), Tibia Vertical (TV), and Next Initial Contact (NIC). Subsequently, an additional 13 intermediate sub-events were selected. Thus, in total, 22 vGRF features were selected as inputs for the WNN. Each vGRF input feature was linked to the corresponding instantaneous measure of the ankle, knee, and hip rotation angles along the sagittal plane. As a result, the 22 vGRF feature inputs were linked to 66 outputs (3 angles $\times$ 22 data points). We collected 300 ground truth data samples in total, from 30 subjects who were asked to perform 10 walking trials each, using a Qualisys motion capture system (Qualisys, Göteborg, Sweden) and Bertec Force Plates of type FP 4060-07 (Bertec Corporation, OH, USA). The data for training and testing the network were selected randomly from the 300 data samples. 70% of the data were used for training (210 samples), while the remainder were used for testing purposes. The hidden layer was modelled using five nodes that were optimized based on the minimum prediction risk principle^16,26^ and was observed to converge to its minimum cost at about 50 epochs. The performance of the WNN was compared with MLP networks, to understand the significance of WNN concept against widely applied MLPs. For the sake of simplicity, the WNN and MLP trained with GI features are labelled as WNN-GI and MLP-GI respectively. MLP-GI was trained with a similar number of hidden layer nodes and training epochs (five hidden layer nodes and 50 training epochs) as WNN-GI, for a fair comparison. A detailed explanation of the methods used in this study is presented under the “Methods” section of this paper.

## Results and discussion

Estimation accuracies were evaluated using the average Pearson Correlation Coefficient (${\overline{\rho }}$) and average Root Mean Square Error ($\overline{{RMSE}}(^\circ )$). ${\overline{\rho }}$ and $\overline{{RMSE}}$ are the common accuracy indicators that were reported in past literature to measure ANN estimation performances. $\rho$ measures the linear relationship between estimated and ground truth angles. *RMSE* measures the difference between the estimated and ground truth angles. The ${\overline{\rho }}$ and $\overline{{RMSE}}(^\circ )$ were calculated by averaging the $\rho$ and *RMSE* scores across the sample set. The network performance was further evaluated based on In-Samples and Out-Samples data. In-Sample accuracies quantify the network’s performance when tested with training data samples. Out-Sample accuracies quantify the network’s generalization ability when tested on data samples that were not used during the training process. The repeatability of accuracies was evaluated with respect to three data groups that were selected using three random sequences (sequence 1, sequence 2, and sequence 3). Each selected input sequence consists of different sets of randomly selected data samples. As such, the network was tested with multiple data groups to investigate whether it can estimate joint angles with similar accuracy levels for any sample group in a given database. The sequence which generated the least ${\overline{RMSE}}$ was considered when evaluating the overall performance of the network. Details on WNN-GI accuracies are listed in Supplementary Information Tables 2–6.

**Figure 2:**
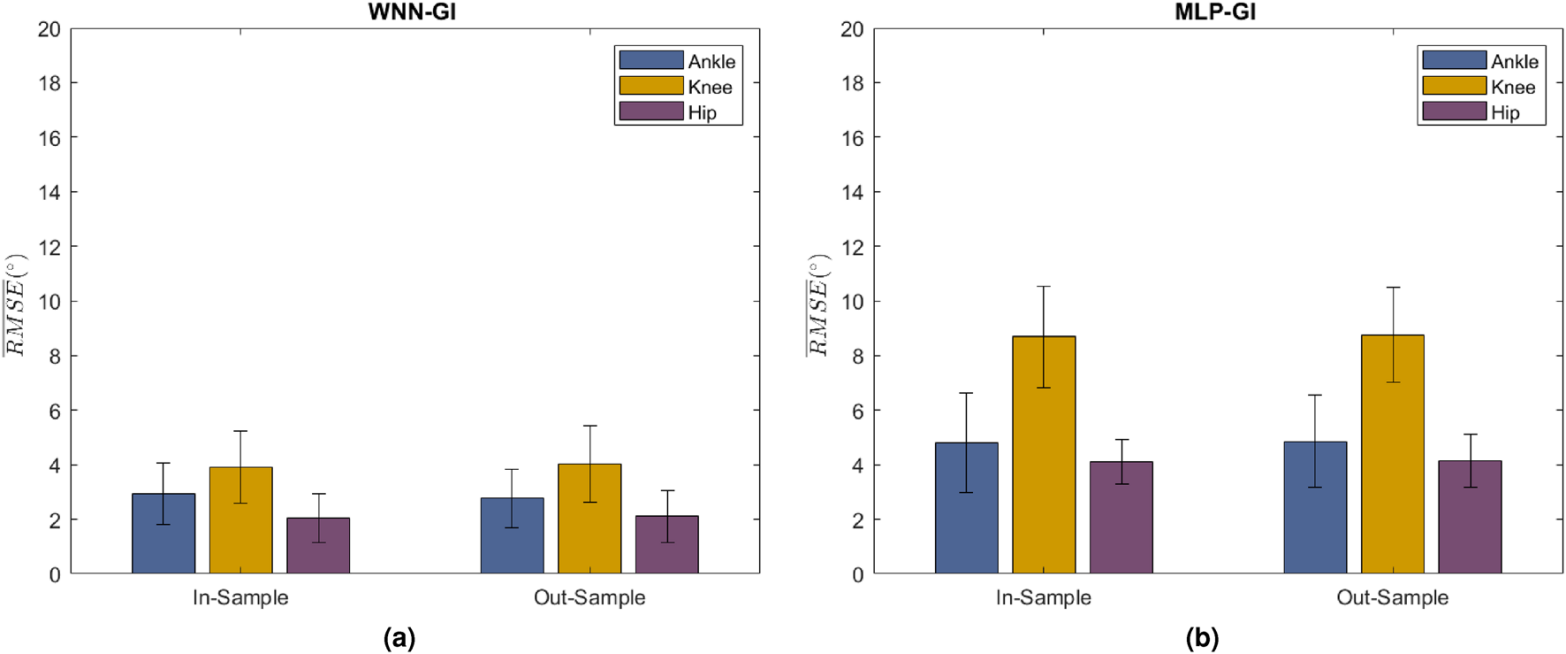
${\overline{RMSE}}$ (${\text {mean}} \pm {\text {standard}}\,{\text{deviation}}$) for In-Sample and Out-Sample estimations corresponding to ankle, knee, and hip angles. (**a**) WNN-GI and (**b**) MLP-GI. Accuracies correspond to the data sequence 1, which reported the least ${\overline{RMSE}}$. All networks are trained with five hidden layer nodes and 50 epochs. More details of accuracies are documented in Tables 2–7, in Supplementary Information.

The ankle, knee, and hip joint angles of WNN-GI were highly correlated with its ground truths reporting ${\overline{\rho }} > 0.945$. ${\overline{RMSE}}$ distributions are indicated in Fig. 2a. The ankle angle ${\overline{RMSE}}$ reported across all samples was $2.860 \pm 1.097^{\circ }$ with an In-Sample and Out-sample accuracy of $2.947 \pm 1.124^{\circ }$ and $2.772 \pm 1.070^{\circ }$ respectively. The knee angle ${\overline{RMSE}}$ reported across all samples was $3.978\pm 1.369^{\circ }$ with an In-Sample and Out-sample accuracy of $3.922\pm 1.326^{\circ }$ and $4.035 \pm 1.411^{\circ }$ respectively. The hip angle ${\overline{RMSE}}$ reported across all samples was $2.071 \pm 0.924^{\circ }$ with an In-Sample and Out-sample accuracy of $2.029 \pm 0.894^{\circ }$ and $2.114 \pm 0.954^{\circ }$ respectively.

**Figure 3:**
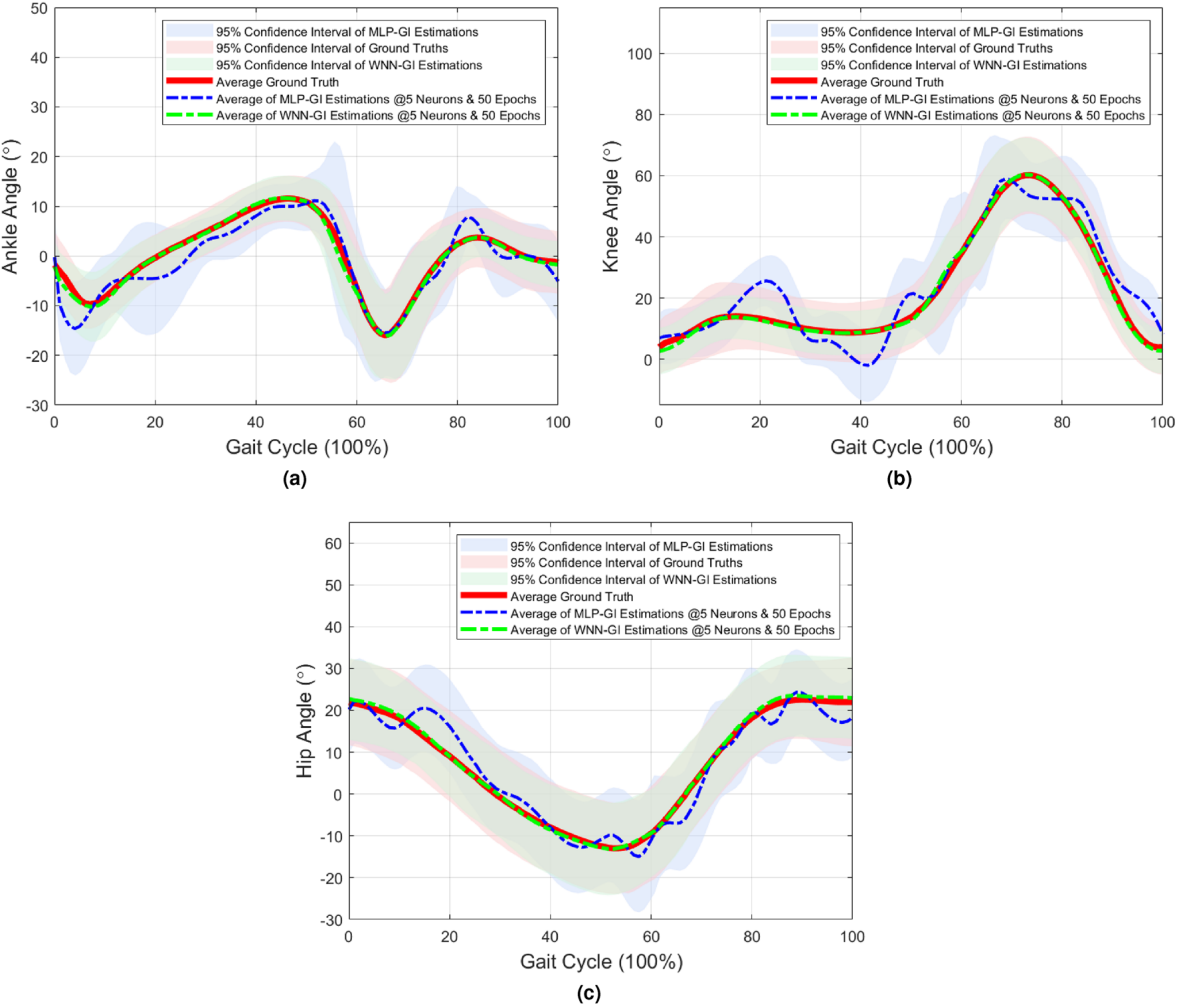
Graphical comparison between average estimated angles of WNN-GI (green dashed line), MLP-GI (blue dashed lines), and the ground truth (red solid line). The green shaded area represents 95% CI of WNN-GI Estimations which is ± 2 standard deviation of the average estimated angle of WNN-GI. The blue shaded area represents 95% CI of MLP-GI Estimations which is ± 2 standard deviation of the average estimated angle of MLP-GI. The red shaded area represents 95% CI of ground truths which is ± 2 standard deviation of the average ground truth. (**a**) Ankle angle (Dorsiflexion is denoted in +ve direction whereas plantar flexion is denoted in −ve direction), (**b**) knee angle (flexion is denoted in +ve direction whereas extension is denoted in −ve direction), and (**c**) hip angle (flexion denoted in +ve direction whereas extension is denoted in −ve direction).

The Out-Sample ${\overline{RMSE}}$s were approximately the same with In-Sample ${\overline{RMSE}}$s, emphasizing the networks’ generalisation ability (Supplementary Information Tables 2–3). WNN-GI network is generalized to estimate accurate joint angles for inputs that were not used during its training process. ${\overline{RMSE}}$s and ${\overline{\rho }}$s reported across all data sequences followed similar levels of accuracy, which indicates the performance repeatability of WNN-GI network (Supplementary Information Table 2).The estimated joint angles and average ground truths were graphically compared to further understand angle characteristics (Fig. 3).

When examined graphically, WNN-GI estimations indicate close fits to its ground truths (Fig. 3). The 95% Confidence Intervals (CI) of the estimated ankle, knee, and hip angles were [16.126 $-24.779$], [72.396 $-4.865$], and [33.245 $-24.104$] respectively. The 95% CI of WNN-GI estimations follow similar trends as the ground truth CI and fall within the normal gait range (Fig. 3). It is also evident that the selective vGRF features of the primary gait events were sufficient for WNN-GI to estimate accurate joint angles. We believe that the efficient network initialization, hidden layer activation using wavelet functions, and GI based feature selection had significantly strengthened WNNs for rapid and accurate estimations.

### Role of data and feature selection

In this study, we focused on improving network performance by reducing the data dimensionality of the input signal. The input data dimensionality was reduced by method GI which focused on selecting only 22 features from the vGRF signal (please refer to “Feature selection” section for an explanation on the feature selection method). These selected 22 features reduced the input data dimensionality by 78% when compared to using a full-length signal (normalised to 100 data points). Currently available methods used sequential inputs, where all the data points of each signal were added one at a time and the network weights were updated once all data points across all samples were added^7–9,17–21,25,27^. Sequential inputs were inserted when the signals are large in dimension. However, the use of sequential inputs requires a larger number of training epochs and hidden layer nodes to accurately learn characteristics of the high dimensional inputs.

### Role of WNN parameter initialization

ANNs are trained by updating the network’s parameters (e.g. weights, biases, and hidden node parameters) until the minimum or the target cost is reached (“Network training and testing” section). Efficient initialization sets the parameters close to the target cost. An efficiently initialized network would require comparatively less time to converge to its target cost. Especially, initializing the translation and dilation parameters is a crucial step towards achieving fast convergence. The hidden layer nodes of the WNN are activated non-linearly by dilating and translating the wavelet activation function. The current constraints encountered in the wavelet function is the sensitivity of parameter initialization associated with its translation and dilation parameters. Wavelet activation functions are closed-loop with limited length and zero average. Therefore, random initialization of translation and dilation parameters might lead to a hidden layer node activation of magnitude zero^16,28^. Wavelon activations with magnitude zero could consequently drag the training time and might cause the network to get stuck at a local cost minimum. Furthermore, random initialization leads to different weight matrices at each training run, resulting in inconsistent outcomes at each training attempt^27^. Therefore, a rigorous fine-tuning process is required to initialize translation and dilation parameters to get an optimum result. These limitations can be avoided by initializing the translation and dilation parameters using a heuristic process (“Network parameter initialization” section). The heuristic process sets the initial parameters of the WNN closer to its target state. As a result, WNNs are less susceptible to be stuck in a local minimum resulting in faster convergence to the target cost. A fast training network will require a comparatively lesser number of training epochs to reach the target cost. The WNN for the underline application reported convergence to its target state at 50 epochs reaching the minimum training cost of 0.0276.

### Role of wavelet activation function

Wavelet activation is the process of using wavelet transform theory to activate the hidden layer nodes while maintaining the universal approximation property of ANNs (“WNN architecture” section). The time series inputs can directly be inserted into the network, where the characteristics will be analysed in the time-frequency domain within the network itself. As a result, the WNN reported a significant reduction in the number of hidden layer units for its function approximation. WNN-GI required only five hidden layer nodes to converge to its optimal state.

Each hidden layer node acts as a wavelet and is activated by the wavelet activation function. A wavelet activation function is a closed-loop waveform with an average value of zero and localized properties. The inputs to the wavelet activation function are non-linearly dilated and translated before its activation. The efficient functionality of the wavelet function depends on the efficient initialization of the translation and dilation parameters. In this study, we have implemented an efficient heuristic method to initialize dilation and translation parameters, making sure the inputs fall within the active bounds of the wavelet function (“Network parameter initialization” section). As a result, hidden nodes that are activated by wavelet functions have higher compression abilities than traditionally used sigmoid activations^15^. When the compression abilities are higher, the learning capability of each hidden layer node gets stronger. Sigmoids are open-loop functions. Hence, the gradient of the sigmoid disappears as its magnitude increases, which consequently slows down the network’s training process. Furthermore, training a randomly initialized network using Gradient Descent algorithm, generate different weight matrices at each training run. These oscillated weights could fluctuate the network gradient, and consequently, lead to an inconsistent convergence rate at each training run.

The overall ${\overline{RMSE}}$ of MLP-GI was $5.964\pm 1.527^{\circ }$. The ${\overline{RMSE}}$ of MLP-GI is higher by 50% when compared with the ${\overline{RMSE}}$ of WNN-GI (See Fig. 2b and Tables 2–3 in Supplementary Information). The patterns of angles estimated by MLP-GI illustrated high fluctuations (Fig. 3 and Table 7 in Supplementary Information). The 95% CI behaviors of MLP-GI estimations fluctuate and fall out of the normal gait range, especially at 20% (Mid Stance), 60% (Toe Off), and 80% (Mid Swing) gait events (Fig. 3). In a normal gait cycle, the main weight bearings take place at the Mid Stance, Toe off and Mid Swing phases, especially at 20%, 60% and 80% of the gait cycle (Fig. 6). It could be seen that the MLP-GI has failed to accurately generate the important joint angle characteristics corresponding to the phases where major changes in the weight bearing occurs. MLP-GI was trained using a similar number of hidden layer units and training epochs as WNN-GI. Poor accuracy of MLP-GI explains that the network has not reached its minimum cost at the $50{\mathrm{th}}$ epoch. Therefore further training is required to reach the minimum cost and achieve accurate joint angle estimations. In contrast, WNNs were capable of fast convergence to the optimal state thus estimating accurate angles with just five hidden layer nodes and 50 training epochs. Fast convergence decreased training duration, whereas the reduced number of hidden layer nodes had simplified the network architecture.

### Comparison with wearable kinematics sensor methods

This paper presents a WNN model that estimates joint angles using selective vGRF inputs measured from the foot. The estimated joint angles are with improved accuracies compared to the existing wearable kinematics sensor-based methods (see Table 1). The ${\overline{RMSE}}$s of the ankle, knee, and hip joint angles were approximately lower by 75%, 53%, and 64%, when compared to a study reported by Cloete et al.^29^ which used 16 wearable inertial sensors for data acquisition. Likewise, the ${\overline{RMSE}}$ of the ankle, knee, and hip angles of our method is approximately lower by 71%, 50%, and 80% respectively, than a seven Inertial Measurement Unit (IMU) method by Tadano et al.^30^. A method by Seel et al.^31^ used six inertial sensors and reported approximately a 43% reduction in ankle ${\overline{RMSE}}$ and 17% reduction in knee ${\overline{RMSE}}$, compared to the accuracies our study. However, Seel’s^31^ method should be further tested using data from multiple subjects, as it was only been validated against data from a single subject. Moreover, Cloete et al.^29^, Tadano et al.^30^ and Seel et al.^31^ focused on attaching multiple wearable kinematics sensors on lower limb segments and joints. Cloette et al.^29^ used 16 sensors, Tadano et al.^30^ used seven sensors each and Seel et al.^31^ utilized six sensors for calculating joint angles of the lower limb. Attachment of multiple kinematics sensors cause discomfort to the wearers and tends to alter their natural walking styles.

Recent research focuses on limiting the number of kinematics sensors to reduce the disadvantages associated with the use of multiple sensors. Though the number of sensors has reduced the accuracies require improvements. In an attempt to reduce the sensors, Hu et al.^32^ used four IMUs to estimate ankle, knee, and hip joint angles. In comparison to Hu et al.^32^, our study reported approximately a 61%, 41%, and 58% reduction in ${\overline{RMSE}}$s of ankle, knee, and hip angles respectively. Sy et al.^33^ documented a recent method which calculated lower limb kinematics using only six wearable inertial sensors. However, in comparison with Sy et al.^33^, our method indicated approximately 60% and 79% reduction in the overall ${\overline{RMSE}}$ error of knee and hip angles respectively. Wearable inertial sensors are affected by drift and sensor offsets that should be removed by advanced signal processing techniques. When compared to the results of the above-mentioned methods that focused on reducing the kinematics sensors, the proposed WNN-GI method reported improved accuracies without relying on any kinematics sensors. ANNs have reported promising results when treating the drift offsets and to achieve better accuracies using a lesser number of wearable kinematics sensors.

Wouda et al.^9^ evaluated ANN’s ability in estimating knee joint angle. In comparison to Wouda et al.^9^, our proposed WNN-GI model reported approximately a 57% overall reduction on the knee ${\overline{RMSE}}$. In a recent comparative study conducted by Figueiredo et al.^34^, the drift error of wearable inertial sensors was reduced using shallow Neural Networks. Our method has significantly reduced the Average Normalized Root-Mean-Square Error (${\overline{NRMSE}}$) of hip angle by approximately 30% than Figueiredo et al.^34^. Moreover, the angles estimated by the proposed WNN-GI method reported the best correlations (${\overline{\rho }}$) when compared to literature in Table 1.

However, WNN-GI method is not applicable for estimating pathological or ageing gait as it was only trained and tested with normal gait from healthy adults. Training a network with healthy adults data and testing it with healthy adults data is practically manageable and would produce accurate estimations. This is because the data characteristics of the training and testing samples are similar in nature. However, once the network is tested with abnormal gait, it will produce inaccurate estimations as it is only been trained with normal gait patterns. On the other hand, training the same network with normal and abnormal gait patterns to estimate joint angles is also not practical due to high variations of data characteristics. It is not recommended to train the same network model to estimate all kinds of data, however, the proposed concept could be expanded to sub-models in identifying different gait patterns and conditions (e.g. estimations of older adults, estimations of gait with arthritis).

**Table 1:** Accuracies of the proposed WNN-GI method versus related literature. Bold font emphasizes the results of the proposed method.

Method	Number of wearable kinematics sensors	Number of subjects	Ankle angle			Knee angle			Hip angle		
			${\overline{RMSE}}(^\circ )$	${\overline{NRMSE}}$ (%)	${\overline{\rho }}$	${\overline{RMSE}}(^\circ )$	${\overline{NRMSE}}$ (%)	${\overline{\rho }}$	${\overline{RMSE}}(^\circ )$	${\overline{NRMSE}}$ (%)	${\overline{\rho }}$
Cloette et al^29^	16	8	11.36	–	0.08	8.47	–	0.89	5.81	–	0.94
Tadano et al.^30^	7	5	9.75	–	0.78	7.88	–	0.97	10.14	–	0.98
Seel et al.^31^	6	1	1.62	–	–	3.30	–	–	–	–	–
Hu et al.^32^	4	8	7.28	–	0.79	6.78	–	0.95	4.91	–	0.97
Sy et al.^33^	6	9	–	–	–	10.00	–	0.87	9.90	–	0.74
Wouda et al.^9^	6	8	–	–	–	9.33	–	0.96	–	–	–
Figueiredo et al.^34^	7	11	–	9.2	0.87	–	6.9	0.94	–	7.9	0.92
**WNN-GI (The proposed method)**	**0**	**30**	**2.86**	**9.80**	**0.95**	**3.98**	**6.78**	**0.98**	**2.07**	**5.54**	**0.99**

The last row is bolded to emphasise the results of the proposed method.

### Significance of the proposed concept

The proposed WNN based joint angle estimation method enabled joint kinematics acquisition without the use of any kinematics sensors and as a result, have simplified the sensor layout as well as have eliminated the requirement of advanced algorithms to treat drift offset. This method relied on foot vGRFs to estimate the complete lower limb joint angle profiles. Foot kinetics are only active during the stance phase of the gait cycle and are less complex compared to kinematics data that focus on multiple joint 3D motions. Subject-specific anthropometric information such as weight, heights, and lengths of segments was not required for the proposed mechanism. Our proposed model acts as a complete solution and sets a future trend to overcome the drawbacks of current available methods. Promising accuracies with the least computational costs make the proposed protocol a suitable real-time estimation model. The inclusion of salient inputs from primary gait events makes the estimation protocol a beneficial tool to be integrated into a smart insole for gait data acquisition.

## Conclusion

In conclusion, this paper presented a WNN model to estimate lower body joint angles using vGRF inputs measured from the foot for gait monitoring applications. vGRF input selection using the knowledge of primary gait events had resulted in accurate joint angle estimations using reduced input data dimensionality. WNNs are prospective ANN models for gait estimations, which reported simpler network architecture using just 5 hidden layer nodes and demonstrated a fast training process with just 50 training epochs while indicating a 50% reduction in its estimation error compared to that of MLP networks. The high accuracies with the least computational cost make WNN a suitable real-time estimation model for gait evaluations. This proposed estimation concept eliminates the requirement of multiple body-mounted sensors for joint motion measurements which instead estimates accurate and complete sagittal plane joint motion profile using inputs from the foot. In comparison to kinematics sensors, foot insoles are easy to attach to the wearer, consist of a lesser complex sensor layout, and not affected by drift sensor offsets. Therefore, the combination of WNNs with descriptive vGRF inputs from primary gait events makes the proposed protocol a beneficial tool to be integrated into smart foot sensor insoles (that generally acquire data based on gait event occurrences) for real-time gait evaluations.

## Future improvements

Instrumented insoles enable foot kinetic measurements in outdoor conditions, easy to attach, comfortable on the wearer and data acquisitions are less complex compared to joint kinematics sensors. The applicability of using foot insoles to estimate joint angles is proven by our earlier study, in which Moticon OpenGo insoles were used to estimate ankle joint angles in the stance phase^35^. The concept proposed in this study has reported promising results in estimating ankle, knee, and hip joint angles across both stance and swing phases using 22 vGRF features measured from the foot. Hence, the proposed model will be extended to a wearable foot insole that has a built-in capability of estimating a full kinematics profile of the lower limb. Transverse and coronal plane joint angles are beneficial to portray the real-life relevance as well as in expanding the clinical and practical value especially in the early identification of gait pathologies^36^. Therefore, in addition to the sagittal plane, this work is expected to be further improved to estimate 3D joint angles.

The proposed model will be expanded into sub-models for estimating different types of walking conditions and gait pathologies. It is not recommended to train the same network for estimating multiple gait types. It can cause confusions when training a network with highly variant data characteristics of different gait types. Therefore, we propose to construct one ANN per gait condition. In our future work, the proposed model will be expanded to sub-models to estimate gait conditions of subjects with different age groups, walking conditions and gait pathologies. The collective set of sub-models together with the simplified sensor layout could further facilitate clinicians in monitoring diverse ranges of gait conditions in outdoor settings.

## Methods

The methodology can be subdivided into three main steps; (1) Data collection & Data Processing, (2) Feature Selection, and (3) Joint angle estimations through WNNs. These steps will be further explained in the upcoming sections.

**Figure 4:**
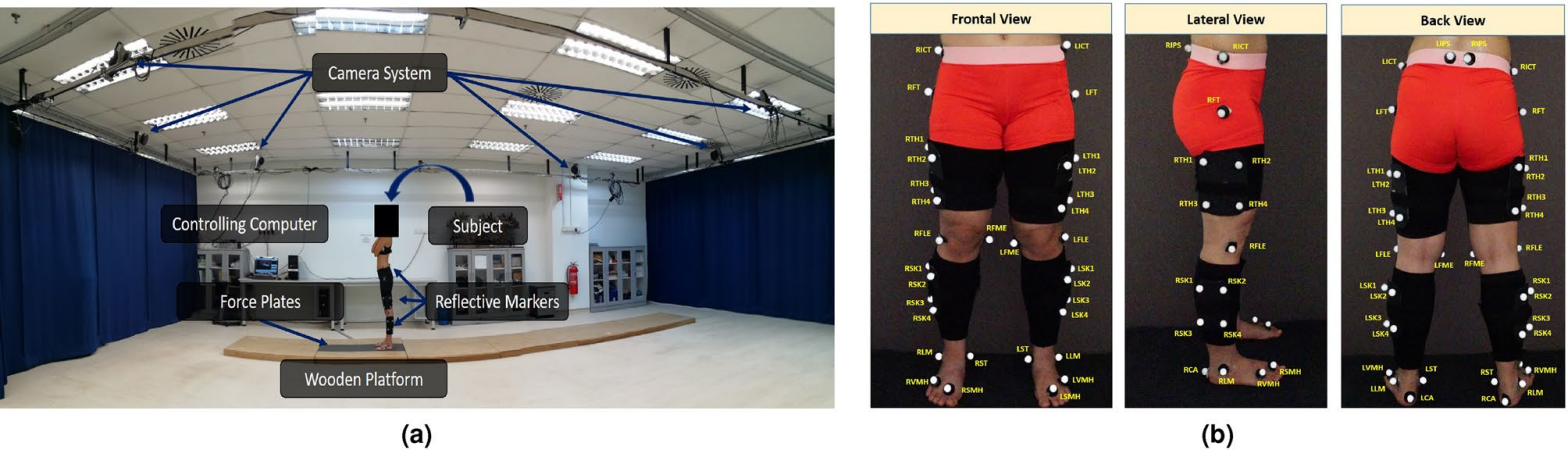
Data Collection Process (**a**) Laboratory setup with six motion cameras (ProReflex MCU, Qualisys, Gothenburg, Sweden) for joint motion measurements, two force plates (Bertec, type FP 4060-07, Bertec Corporation, OH, USA) embedded on to a 10m long wooden platform for vGRF measurements. (**b**) Marker placement with 36 markers recommended by Visual3D (C-motion Inc., Kingston, Canada). Double-sided adhesive stickers were used to stick markers on the segments and joints. Cluster markers with four individual markers were attached to each shank and thigh segments. Cluster markers were tighten using a stretchable band, to ensure no errors due to loosening attachments. All markers are labelled with a unique marker name.

## Data collection and processing

Data was collected from 30 young adults with no history of lower extremity injuries, surgery and gait disorders (age = $22.48 \pm 2.09$ years, height = $1.67 \pm 0.10$ m, mass =$63.74 \pm 12.48$ kg, and Body Mass Index (BMI) = $22.51 \pm 3.26$ kg/${\text {m}}^2$). This study was conducted in accordance with the Declaration of Helsinki as well as in agreement and approval with the Human Research Ethics Committee of Monash University (MUHREC). Subjects were informed about the experimental procedure and informed written consent was obtained from each subject before data collection. Informed written consent was also obtained from subjects who appear on figures, for publication of identifying information and images in an online open-access publication. All subjects were free of any injuries or orthopaedic disorders.

**Figure 5:**
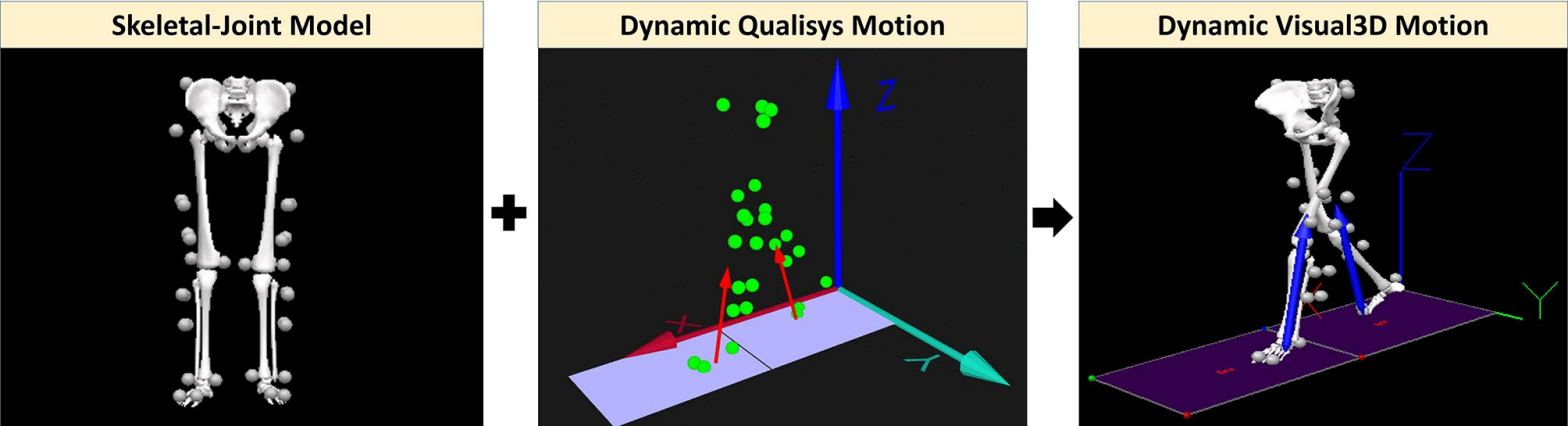
Visual3D data digitizing process in three steps namely; 1. Construction of the static skeletal-joint model, 2. Insertion of the dynamic Qualisys motion files to the static skeletal-joint model which transforms its static motion to dynamic motion, and 3. Extraction of gait parameters through analysing the motion of the dynamic skeletal-joint model. The gray and green circles denote the attached markers. The vertical arrows denoted the vGRFs measured from the force plates. The two force plates are depicted in squares. The images of Skeletal-Joint mModel and Dynamic Visual3D motion, were screen captured from Visual3D Lite v4.96.11^37^ (C-motion Inc., Kingston, Canada). Dynamic Qualisys Motion image was screen captured from the Qualisys Track Manager 2.6^38^ (Qualisys, Göteborg, Sweden).

**Figure 6:**
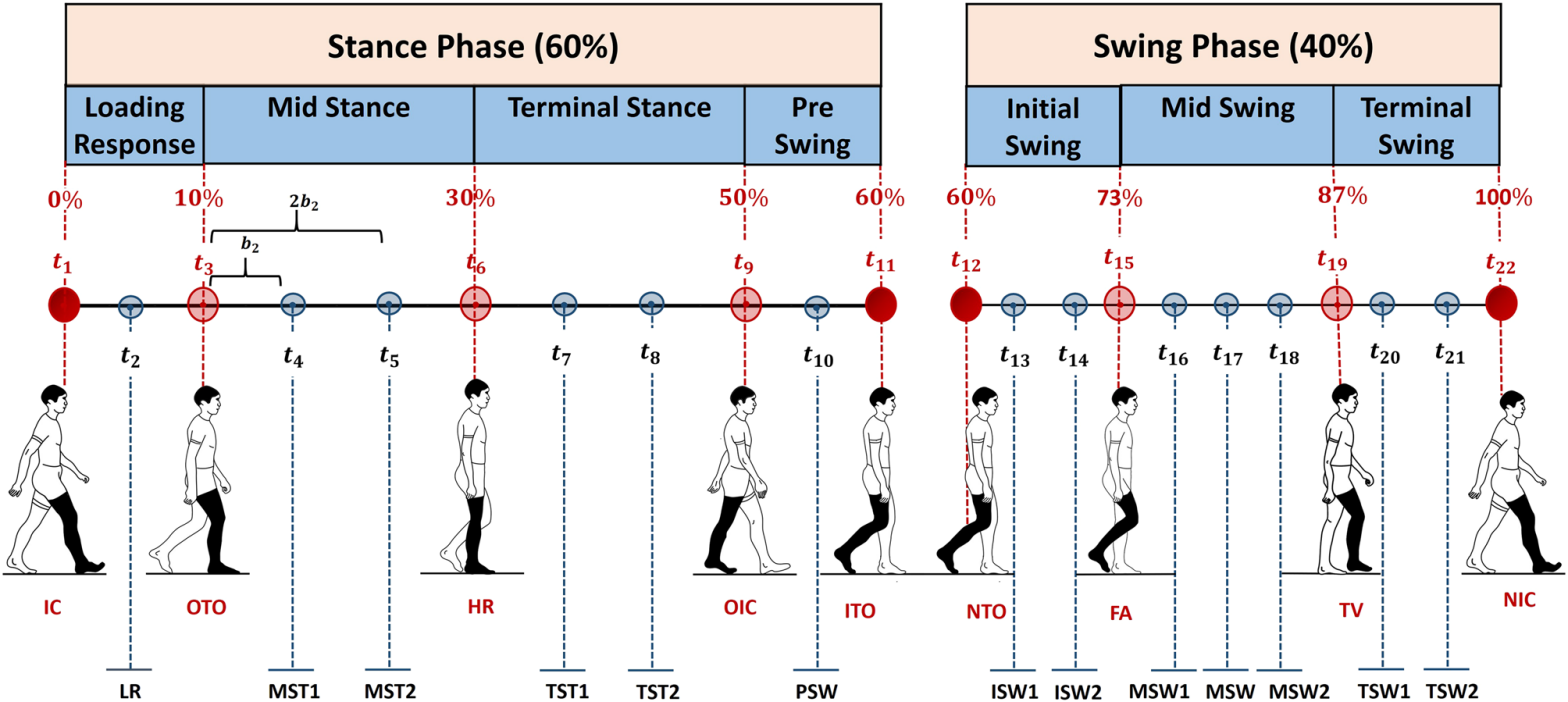
Gait event occurrence indexes used in the GI feature selection process. 22 gait events are used in the feature selection process. Each index is labelled based on its event number (e.g. $t_{1}$ for event 1 and $t_{22}$ for event 22). Gait indexes of main events are marked in red (bigger) circles, where the IC, ITO, NTO, and NIC indexes extracted from Visual3D (C-motion Inc., Kingston, Canada) automatic event detection algorithm are marked in red (bold) circles. The gait indexes of intermediate events are marked in blue (small) circles. The pre-defined gait indexes are indicated in percentages of the gait cycle. Each intermediate event indexes are calculated by shifting the starting gait index of the sub-phase by a step size $b$ (see Eq. 1). The number of steps taken to locate the index of each intermediate event is a multiple of the intermediate event number of the sub-phase. For example, this figure illustrated the step size ($b_{2}$) taken in extracting indexes within $2{\mathrm{nd}}$ sub-phase (Mid Stance). The index ($t_{4}$) correspond to the first intermediate event of $2{\mathrm{nd}}$ sub-phase was extracted by taking a step size of $b_{2} \times 1$, whereas the index $t_5$ corresponding to the $2{\mathrm{nd}}$ intermediate event was extracted by taking a step size of $b_{2} \times 2$.

Joint motion was measured using six motion cameras (ProReflex MCU, Qualisys, Gothenburg, Sweden) and foot kinetics were measured using two force plates (Bertec). The force plates were embedded into a wooden platform. Data were sampled at a 100 Hz frame frequency. Subjects were asked to walk barefoot at their self-selected walking speed on a 10-meter long wooden platform. The complete laboratory setup is depicted in Fig. 4a. Cameras captured movements based on reflective markers placed on body segments and joints. In this study, a clinical marker set with 36 markers recommended by Visual3D was adopted, as depicted in Fig. 4b.

Dynamic trails (walking) were recorded repeatedly ten times for each subject. Overall, 300 gait samples were acquired (30 subjects $\times$ 10 gait trials). Measured data were digitized in Visual3D Motion Capture Data Analysis software to extract vGRFs, joint angles, as well as gait timings of IC, NIC, ITO and NTO.

Gait parameters were calculated by transforming the dynamic Qualisys motion files to the Visual3D static skeletal-joint model. Figure 5 illustrates the data digitizing process. Angles were extracted with respect to the sagittal plane. Hip flexion/extension was calculated using trunk and thigh segments. Knee flexion/extension, was determined from the thigh and shank segments. Ankle plantar/dorsiflexion was computed by shank and foot segments. vGRFs were computed from analogue signals of the force plates. The extracted gait timings $t_{IC}$, $t_{NIC}$, $t_{ITO}$, and $t_{NTO}$ correspond to events IC, NIC, ITO, and NTO respectively. $t_{IC}$, $t_{NIC}$, $t_{ITO}$, and $t_{NTO}$ were used for partitioning the gait signal into a single gait cycle (from IC to NIC or ITO to NTO). All measured data samples were filtered using a $1{\mathrm{st}}$ order Butterworth low pass filter with $-3$DB cut off frequency, determined using the Fast Fourier Transform spectrum. Next, feature selection was applied to reduce the data dimensionality.

**Figure 7:**
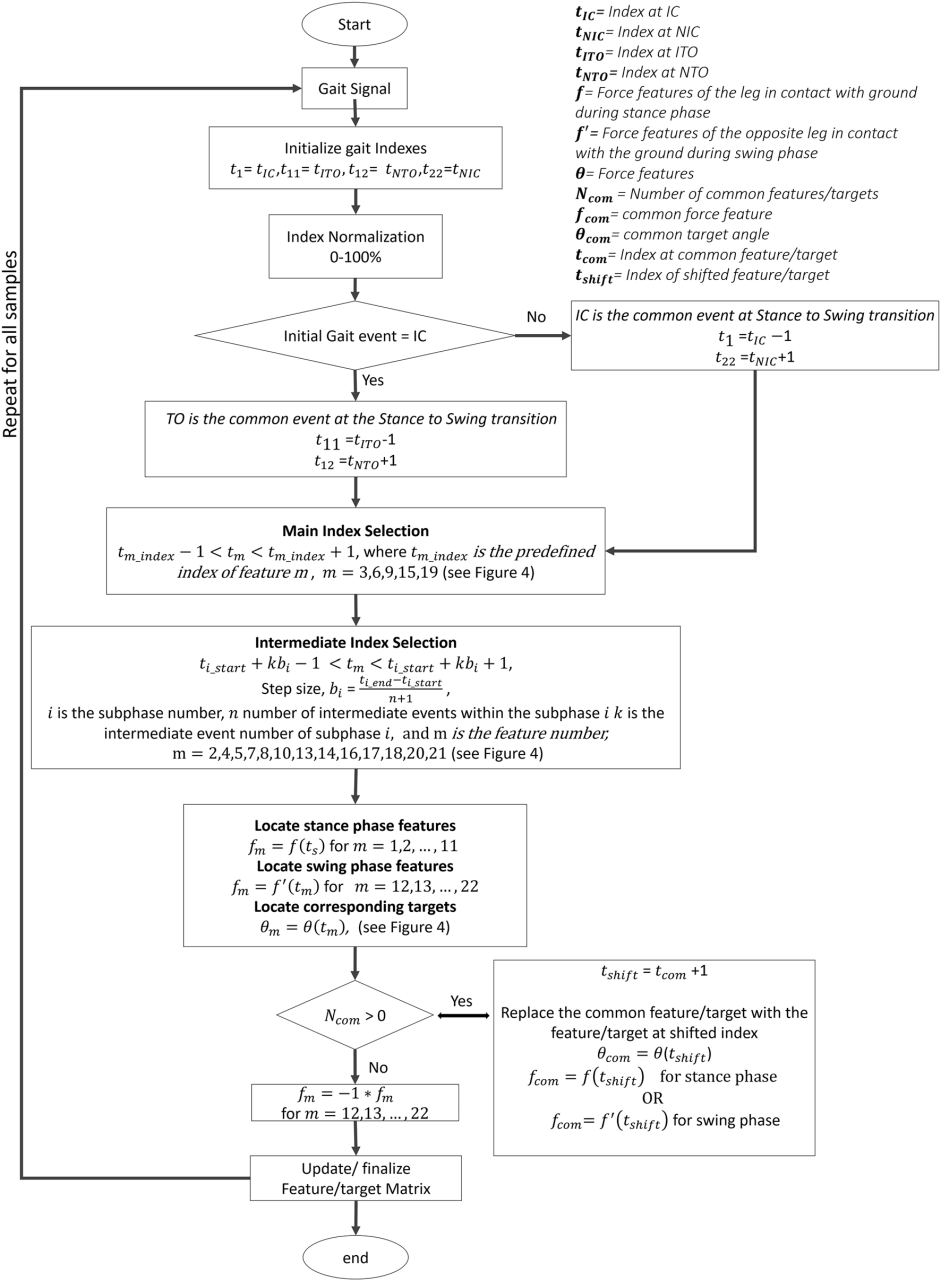
GI feature selection process. The gait indexes $t_{IC}$, $t_{NIC}$, $t_{ITO}$, and $t_{NTO}$ were extracted from Visual3D (C-motion Inc., Kingston, Canada). The index range is normalised to 0–100%. The common event between stance and swing phases (IC or TO) is identified and shifted by $\pm 1\%$. The indexes of main and intermediate events as well as its corresponding features and targets are extracted. Any existing common feature is identified, and its index was shifted by +1% to extract an adjacent feature. Swing phase features were multiplied by a constant negative factor ($-1$) to avoid clashes with patterns in the stance phase. Features and targets were organised in the matrix format. The feature selection process was repeated for all samples.

## Feature selection

Data with reduced dimensionality enable fast training and improves network performance^39,40^. In this study, we present method GI, which reduces data dimensionality by selecting salient points of the signal based on prior knowledge of gait intervals ($t$). Selected vGRF inputs are referred as features. Stance phase features are denoted as $f$ and swing phase features are denoted as $f'$. During the swing phase, the foot stays off the ground and the vGRFs of the swing leg are zero-valued. While the swing foot stays off the ground, the opposite foot is in the stance phase. During the stance phase, the foot stays in contact with the ground and produces non-zero valued vGRFs. Therefore, swing leg vGRF features are selected with respect to the opposite leg vGRF ($f'$) signal. This process provides a unique set of features, as it avoids the constant zero valued vGRFs of the swing leg. Angles are referred as targets and are denoted by $\theta$. Method GI selected 22 vGRF features with respect to gait intervals of 22 events. Figure 6 illustrates the gait interval occurrences of 22 events. The feature selection steps are depicted in Fig. 7. The algorithm is coded in Matlab R2019a (MathWorks, USA).

Nine features are chosen using gait intervals of nine main events namely; IC, OTO, HR, OIC, ITO, NTO, FA, TV, and NIC. Gait event indexes of IC, ITO, NTO, and NIC were extracted from the Visual3D automatic event detection pipeline. The rest of the main gait events were selected based on pre-defined gait indexes, bounded by a ± 1% buffer interval^41,42^. Method GI first located its gait index and next extracted its corresponding vGRF features $f(t)$, $f'(t)$, and relative angles $\theta (t)$. Before the selection process, the index range of the partitioned gait signals was normalised to 0–100%. Next, the algorithm removed existing common events that repeatedly occurred through the gait cycle. For example, when a gait cycle is initiated with a stance phase, ITO and NTO will be the common events at the stance to swing transition point. When the gait cycle is initiated with the swing phase, IC and NIC would be the common events at the transition point between the swing and stance phase. Hence, before selecting features, the common event between stance and swing phases were identified. The index of the identified common event is shifted by $\pm 1\%$ to extract pre and post events from the common index point ($t_{IC}-1$ and $t_{NIC}+1$ or $t_{ITO}-1$ and $t_{NTO}+1$). These pre and post indexes points to two distinct neighbouring events of the identified common point.

The remaining 13 features were selected with respect to gait intervals of intermediate events namely; Loading Response (LR), two intermediate events within Mid Stance (MST1 and MST2), two intermediate events within Terminal Stance (TST1 and TST2), Pre Swing (PSW), two intermediate events within Initial Swing (ISW1 and ISW2), three intermediate events within Mid Swing (MSW1, MSW, and MSW2), and two intermediate events within Terminal Swing (TSW1 and TSW2). Each sub-phase acts as a transition period from one gait event to another. As a result, vital changes in weight bearings occurs during these sub-phases. Hence, we considered events within each of these sub-phases to extract important insights of the signal. Intermediate event indexes were tracked using defined step sizes. Step size, $b_i$, is the gap between the starting and ending gait event indexes which was calculated by Eq. (1). Each intermediate event was located by taking steps from the starting gait event index ($t_{i\_start}$) of the sub-phase. The number of steps needed to locate an event is proportional to the event number within each sub-phase.

1 $$\begin{aligned} b_i=\frac{t_{i\_end}-t_{i\_start}}{n+1} \end{aligned}$$

where $i$ is the sub phase number, $t_{i\_start}$ and $t_{i\_end}$ are the indexes of starting and ending events of the sub phase $i$. $n$ is the number of intermediate events of sub phase $i$.

2 $$\begin{aligned} (t_{i\_start}+kb_i)-1 \le t_{m} \le (t_{i\_start}+kb_i)+1 \end{aligned}$$

where $m$ is the feature number and $m=2,4,5,7,8,10,13,14,16,17,18,20,21$. $k$ is the intermediate event number within sub-phase $i$.

Table 1 in Supplementary Information explains the heuristic of selecting indexes $t$. After the selection of features, the algorithm further searched for any common features and if identified the index of common feature is shifted by +1% until all common features were removed. The swing phase features ($f_{m=12...22}$) were multiplied by a constant negative factor ($-1$) to avoid clashes with similar features in the stance phase. At the final step, the selected features and targets are organised into matrix form to be used with ANNs. Equations (3) and (4) depicts the structure of input features (*x*) and its target outputs (*y*) matrices used in ANN training.

3 $$\begin{aligned} Input\ Features= & {} x= \begin{bmatrix} f_{1,1} &{}\quad f_{1,2} &{}\quad \dots &{}\quad f_{1,n} \\ f_{2,1} &{}\quad f_{2,2} &{}\quad \dots &{}\quad f_{2,n} \\ \dots &{}\quad \dots &{}\quad \dots &{}\quad \dots \\ f_{m,1} &{}\quad f_{m,2} &{}\quad \dots &{}\quad f_{m,n} \end{bmatrix} \end{aligned}$$

4 $$\begin{aligned} Target\ Outputs= & {} y= \begin{bmatrix} \theta ^{a}_{1,1} &{}\quad \theta ^{a}_{1,2} &{}\quad \dots &{} \quad \theta ^{a}_{1,n} \\ \theta ^{a}_{2,1} &{}\quad \theta ^{a}_{2,2} &{}\quad \dots &{}\quad \theta ^{a}_{2,n} \\ \dots &{}\quad \dots &{}\quad \dots &{}\quad \dots \\ \theta ^{a}_{m,1} &{}\quad \theta ^{a}_{m,2} &{}\quad \dots &{}\quad \theta ^{a}_{m,n} \\ \\ \theta ^{k}_{1,1} &{}\quad \theta ^{k}_{1,2} &{}\quad \dots &{}\quad \theta ^{k}_{1,n} \\ \theta ^{k}_{2,1} &{}\quad \theta ^{k}_{2,2} &{}\quad \dots &{}\quad \theta ^{k}_{2,n} \\ \dots &{}\quad \dots &{}\quad \dots &{}\quad \dots \\ \theta ^{k}_{m,1} &{}\quad \theta ^{k}_{m,2} &{}\quad \dots &{}\quad \theta ^{k}_{m,n} \\ \\ \theta ^{h}_{1,1} &{}\quad \theta ^{h}_{1,2} &{}\quad \dots &{}\quad \theta ^{h}_{1,n} \\ \theta ^{h}_{2,1} &{}\quad \theta ^{h}_{2,2} &{}\quad \dots &{}\quad \theta ^{h}_{2,n} \\ \dots &{}\quad \dots &{}\quad \dots &{}\quad \dots \\ \theta ^{h}_{m,1} &{} \quad \theta ^{h}_{m,2} &{}\quad \dots &{}\quad \theta ^{h}_{m,n} \end{bmatrix} \end{aligned}$$

where vGRF features are denoted as $f$. The ankle, knee, and hip target angles are denoted as $\theta ^{a}$, $\theta ^{k}$, and $\theta ^{h}$ respectively. The columns represent the samples ($n$). The rows in Eq. (3) represent features ($m$). The rows in Eq. (4) represent the targets ($3\times m$). $n=300$ is the total number of samples, $m=22$ is the total number of features. Selected features ($x$) and its corresponding targets ($y$) were then utilized for training and testing the ANN model.

## Joint angle estimations through WNNs

### WNN architecture

**Figure 8:**
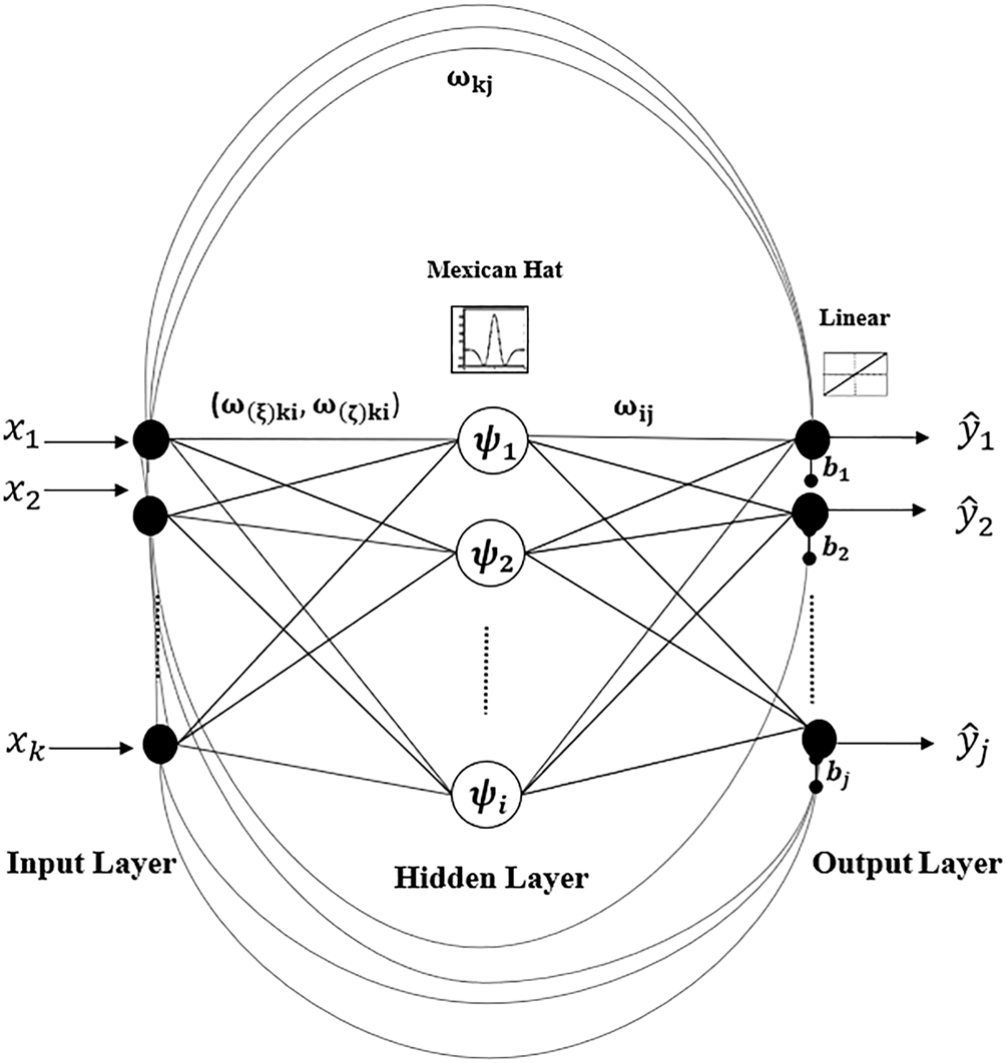
WNN architecture. The WNN consists of two layers namely; input layer, hidden layer, and output layer. $k$, $i$, and $j$ denote the number of input, hidden, and output nodes respectively. The $\omega _{kj}$ is the weight between input node $k$ and output node $j$. Similarly, $\omega _{ij}$ is the weight between hidden layer node $i$ and output node $j$. $\omega _{(\xi )ki}$ is the translation and $\omega _{(\varsigma )ki}$ is the dilation between hidden layer node $i$ and input node $k$. $b_{j}$ is the bias of $j{\mathrm{th}}$ output node. The hidden layer nodes are activated by the Mexican hat wavelet function. The output layer nodes are activated using the linear activation function.

The structure of a two-layered WNN^16^ is illustrated in Fig. 8. WNN learns the relationship between inputs and outputs through learning the data patterns of the given set of examples iteratively by self-adjusting networks’ parameters ($w$) until it converges to the desired target state^43^. The parameter vector ($w$) of the WNN network comprises:

5 $$\begin{aligned} \omega =(\omega _{kj}, \omega _{ij}, \omega _{(\xi )ki}, \omega _{(\varsigma )ki},b_{j}) \end{aligned}$$

$\omega _{kj}$ is the weight between input node $k$ and output node $j$. $\omega _{ij}$ is the weight between hidden layer node $i$ and output node $j$. $\omega _{(\xi )ki}$ is the translation and $\omega _{(\varsigma )ki}$ is the dilation between hidden layer node $i$ and input node $k$. $b_{j}$ is the bias of $j{\mathrm{th}}$ output node.

WNNs modelled the relationship between input and output variables by considering its linear and non-linear aspects separately. The linear relationships between inputs and outputs were modulated by adding a direct connection between input and output layers^16^. Direct connection maps data relationships using a linear activation function. The non-linear relationships between inputs and outputs were modulated within the hidden layer. Hidden layer nodes, which are also known as wavelons, decomposed inputs from time to frequency domain. Data points that enter the hidden layer nodes are non-linearly activated by translating and dilating by the mother wavelet function. These transformed data points were then linearly activated at the output layer and the bias terms ($b_{j}$) were added, generating desired outputs. The bias terms were added to handle the non-zero mean. This study constructed a WNN with 22 input nodes and 66 output nodes corresponding to 22 features and 66 targets selected based on GI. Network output is given by the following expression in Eq. (6):

6 $$\begin{aligned} {\hat{y}}_{j}= \sum _{j=1}^{N^{o}} \psi _{i}({\mathbf{x}} ) \omega _{ij} + \sum _{k=1}^{N^{l}} x_{\textit{k}} \omega _{kj}+b_{j} \end{aligned}$$

Each hidden layer node was activated by the wavelet activation function. $\psi _{i}({\mathbf{x}} )$ is a multidimensional wavelet, constructed by the product of $N^{l}$ scalar wavelets. ${\mathbf{x}}$ is the input vector. The multidimensional wavelets were computed by Eq. (7):

7 $$\begin{aligned} \psi _{i}({\mathbf{x}} )=\prod _{k=1}^{N^{l}} \psi (Z_{ki}) \end{aligned}$$

$Z_{ki}$ denotes the translated and dilated version of input $x_{ki}$. $Z_{ki}$ was computed by Eq. (8):

8 $$\begin{aligned} Z_{ki}= \frac{ x_{i} - \omega _{(\xi )ki} }{\omega _{(\varsigma )ki}} \end{aligned}$$

$\psi$ is the mother wavelet. The selection of the mother wavelets depends on the application. For this study, we used Mexican Hat, given by Eq. (9). Mexican Hat mother wavelet function was proven to perform well in various applications and been suggested in a recent review by Alexandridis et al.^16,44^. Mexican Hat is a real-valued symmetric function that was reported to perform well in identifying peaks and valleys of non-linear data structures^45^. Hence, Mexican Hat wavelet function would be an ideal choice in capturing non-linear patterns of vGRFs and joint angles.

9 $$\begin{aligned} \psi (Z_{ki})= (1-Z_{ki}^{2})e^{-\frac{1}{2}Z_{ki}^{2}} \end{aligned}$$

At the output layer, the weighted sum of linearly activated $\psi _{i}({\mathbf{x}} ) \omega _{ij}$ and the weighted sum of linear activated $x_{\textit{k}} \omega _{kj}$ were calculated and a bias term $b_{j}$ was added, generating the final network output, as indicated in Eq. (6).

### Network parameter initialization

Weights between the nodes ($\omega _{kj}$ and $\omega _{ij}$) were initialised to random values^16^. Bias ($b_{j}$) was initialized to the mean of target outputs calculated across all samples. Initialization of translation and dilation parameters ($\omega _{(\xi )ki}$ and $\omega _{(\varsigma )ki}$) were performed using a heuristic method^16,46^. Parameters were initialized based on the input domain of training samples^47^. The heuristic initialization process is indicated in Eqs. (10)–(13).

10 $$\begin{aligned} \omega _{(\xi )ki}= & {} 0.5(N_{k}+M_{k}) \end{aligned}$$

11 $$\begin{aligned} \omega _{(\varsigma )ki}= & {} 100(N_{k}-M_{k}) \end{aligned}$$

where $\omega _{(\varsigma )ki} \le 1$. $M_{k}$ and $N_{k}$ are defined as the maximum and minimum of input $x_{k}$;

12 $$\begin{aligned} M_{k}= & {} {\max }_{p=1,\dots ,N}(x_{kp}) \end{aligned}$$

13 $$\begin{aligned} N_{k}= & {} {\min }_{p=1,\dots ,N}(x_{kp}) \end{aligned}$$

where *N* defines the total number of samples.

### Number of hidden layer nodes

In this study, the number of hidden layer nodes was selected based on the minimum prediction risk principal^16,26^. Prediction risk is the anticipated performance of the network on testing data and is given by Eq. (14):

14 $$\begin{aligned} {\text {Prediction}}\,{\text{ Risk}}=\frac{1}{N^{tst}} \sum _{p=1}^{N^{tst}} ({y}^{*}_{p}- {\hat{y}}^{*}_{p})^{2} \end{aligned}$$

where $y^{*}_{p}$ is the target output and ${\hat{y}}^{*}_{p}$ is the network output of the testing sample $p$. $N^{tst}$ is the total number of testing samples. For this study, $N^{tst}$is set to 90 samples. The number of hidden layer nodes that generated minimum prediction risk was chosen. In accordance with the minimum prediction risk reported, five hidden layer nodes are utilized for the WNN proposed in this study.

### Network training and testing

After initialization, the WNN was trained by updating each parameter $\omega$ while minimizing its cost function using gradient descent with momentum backpropagation algorithm^48^. Training is performed repeatedly by updating the network parameters ($\omega$) until the cost ($L$, see Eq. (15)) achieved its minimum ($L_{min}$) or epochs reached its maximum ($E_{max}$).

15 $$\begin{aligned} {\text {Cost}}= L =\frac{1}{N^{trn}} \sum _{p=1}^{N^{trn}} ({y}_{p}- {\hat{y}}_{p})^{2} \end{aligned}$$

where $y_{p}$ is the target output and ${\hat{y}}_{p}$ is the network output of the training sample $p$. $N^{trn}$ is the total number of training samples.

The fixed lower bound of cost ($L_{min}$) was set to 0.00001. Initial training epoch ($E$) was set to one and incremented with steps of one. $E_{max}$ was set to 50. The training performance was evaluated at the $50{\mathrm{th}}$ epoch to make validate whether the WNN reached its target state, or else the network was further trained till its optimal state was reached. Samples were shuffled and randomly grouped for training and testing purposes. The random selection process was repeated three times to yield three groups (sequences) of input data namely; (1) sequence 1, (2) sequence 2, and (3) sequence 3. The network was trained and tested with the three data sequences, to investigate the repeatability of networks’ estimation performance. 70% (equivalent to 210 samples) of shuffled data samples were used for training ($N^{trn}$). The remaining 30% samples (equivalent to 90 samples) were used for testing ($N^{tst}$).

The network’s output signal was a combination of ankle, knee, and hip angle points. Firstly ankle, knee, and hip angle points were separately extracted. Next, the complete angle profile was reconstructed using the Spline Interpolation method^49^. Accuracies are calculated by comparing the reconstructed angle profiles with the measured ground truth. Accuracies were reported in $\overline{{RMSE}}(^\circ )$ and ${\overline{\rho }}$. Besides, average NRMSE ($\overline{{NRMSE}}(\%)$) was also calculated to compare WNN-GI with existing literature that used $\overline{{NRMSE}}$ as their accuracy measure. $\overline{{RMSE}}(^\circ )$, ${\overline{\rho }}$ and $\overline{{NRMSE}}$ were calculated by taking average of $RMSE$, $\rho$ and $NRMSE$ values across the data samples. WNN-GI performance was also evaluated with respect to In-Samples and Out-Samples. In-Samples are the data samples that were used during the network training, whereas Out-Samples are new data samples that were not used for the network’s training. Accuracies were elaborated into each angle type (ankle, knee, and hip) across three data input sequences (sequence 1, sequence 2, and sequence 3). The sequence that generated the least average ${\overline{RMSE}}$ was chosen. According to the selected sequence, average ${\overline{RMSE}}$ of three angles (ankle, knee, and hip) were calculated considering all estimation (including both In-Samples and Out-Samples).

## Supplementary Information

SupplementaryInformation 1.

Supplementary Information 2.
