# Supplementary material for: Joint angle estimation with wavelet neural networks
Source: Sci Rep. 2021 May 13;11:10306. doi: 10.1038/s41598-021-89580-y (PMC8119494; doi:10.1038/s41598-021-89580-y)
Supplement: Supplementary file 1 — SupplementaryInformation 1. [file 41598_2021_89580_MOESM1_ESM.pdf]

Joint Angle Estimation with Wavelet Neural Networks

—

SUPPLEMENTARY INFORMATION

Saaveethya Sivakumar[1,2,*], A.A Gopalai[1,*], King Hann Lim[2], D. Gouwanda[1], and S. Chauhan[3]

[1]Monash University Malaysia, School of Engineering
[2]Curtin University Malaysia, Department of Electrical and Electronic Engineering
[3]Monash University Australia, Department of Mechanical & Aerospace Engineering
[*]saaveethya@ieee.org, alpha.agape@monash.edu

ABSTRACT

This paper presents a Wavelet Neural Network (WNN) based method to reduce reliance on wearable kinematic sensors in gait analysis. Wearable kinematic sensors hinder real-time outdoor gait monitoring applications due to drawbacks caused by multiple sensor placements and sensor offset errors. The proposed WNN method uses vertical Ground Reaction Forces (vGRFs) measured from foot kinetic sensors as inputs to estimate ankle, knee, and hip joint angles. Salient vGRF inputs are extracted from primary gait event intervals. These selected gait inputs facilitate future integration with smart insoles for real-time outdoor gait studies. The proposed concept potentially reduces the number of body-mounted kinematics sensors used in gait analysis applications, hence leading to a simplified sensor placement and control circuitry without deteriorating the overall performance.

This document contains four supplementary tables in which Table.1 describes the heuristic of GI feature selection method. Table.2 presents an overall summary of WNN-GI and MLP-GI accuracies. Table.3 is a summary of In-Sample, Out-Sample and All Sample $\overline{RMSE}(^\circ)$ accuracies with respect to each angle type of WNN-GI and MLP-GI. Table.4 is a summary of In-Sample, Out-Sample and All Sample $\overline{\rho}$ accuracies with respect to each angle type of WNN-GI and MLP-GI. Table.5 is a summary of In-Sample, Out-Sample and All Sample $\overline{NRMSE}(\%)$ accuracies with respect to each angle type of WNN-GI and MLP-GI. Table.6 and Table.7 describes the $\overline{RMSE}(^\circ)$ and $\overline{\rho}$ results across all three data sequences respect to WNN-GI and MLP-GI respectively.

1 Supplementary- GI Feature Selection

Feature/Target Number	Gait Event abbreviation	Gait Index	Buffer
**1**	**IC***	t_1	-
2	LR	$t_1+b_1-1 \leq t_2 \leq t_1+b_1+1$	$b_1=\frac{t_3-t_1}{2}$
**3**	**OTO**	$9 \leq t_3 \leq 11$	-
4	MST1	$t_3+b_2-1 \leq t_4 \leq t_3+b_2+1$	$b_2=\frac{t_6-t_3}{3}$
5	MST2	$t_3+2b_2-1 \leq t_5 \leq t_3+2b_2+1$	
**6**	**HR**	$t_6=29 \leq t \leq 31$	-
7	TST1	$t_6+b_3-1 \leq t_7 \leq t_6+b_3+1$	$b_3=\frac{t_9-t_6}{3}$
8	TST2	$t_6+2b_3-1 \leq t_8 \leq t_6+2b_3+1$	
**9**	**OIC**	$34 \leq t_9 \leq 36$	-
10	PSW	$t_9+b_4-1 \leq t_{10} \leq t_9+b_4+1$	$b_4=\frac{t_{11}-t_9}{2}$
**11**	**ITO***	t_{11}	-
**12**	**NTO***	t_{12}	-
13	ISW1	$t_{12}+b_5-1 \leq t_{13} \leq t_{12}+b_5+1$	$b5=\frac{t_{15}-t_{12}}{3}$
14	ISW2	$t_{12}+2b_5-1 \leq t_{14} \leq t_{12}+2b_5+1$	
**15**	**FA**	$t_{15}=72 \leq t \leq 74$	-
16	MSW1	$t_{15}+b_6-1 \leq t_{16} \leq t_{15}+b_6+1$	$b_6=\frac{t_{19}-t_{15}}{4}$
17	MSW	$t_{15}+2b_6-1 \leq t_{17} \leq t_{15}+2b_6+1$	
18	MSW2	$t_{15}+3b_6-1 \leq t_{18} \leq t_{15}+3b_6+1$	
**19**	**TV**	$t_{19}=86 \leq t \leq 88$	-
20	TSW1	$t_{19}+b_7-1 \leq t_{20} \leq t_{19}+b_7+1$	$b_7=\frac{t_{22}-t_{19}}{3}$
21	TSW2	$t_{19}+2b_7-1 \leq t_{21} \leq t_{19}+2b_7+1$	
**22**	**NIC***	t_{22}	-

Supplementary Table. 1. Method GI-heuristic of feature Selection. The rows marked in Bold text corresponds to heuristics of main events. Events marked by (*) are extracted from Visual3D automatic gait event detection algorithm.

2 Supplementary- Results

Accuracy Measure	WNN-GI	MLP-GI
In-Sample $\overline{RMSE}(^\circ)$	2.966 ± 1.129	5.982 ± 1.605
Out-Sample $\overline{RMSE}(^\circ)$	2.974 ± 1.161	5.946 ± 1.527
All Samples $\overline{RMSE}(^\circ)$	2.970 ± 1.145	5.964 ± 1.567
Minimum $\overline{\rho}$	0.945	0.846

Supplementary Table. 2. Overall joint angle estimation accuracies of WNN-GI and MLP-GI networks.

**WNN-GI** $\overline{RMSE}(^\circ)$			
**Angle**	**In-Sample**	**Out-Sample**	**All Samples**
Ankle	2.947 ± 1.124	2.772 ± 1.070	2.860 ± 1.097
Knee	3.922 ± 1.326	4.035 ± 1.411	3.978 ± 1.369
Hip	2.029 ± 0.894	2.114 ± 0.954	2.071 ± 0.924
**MLP-GI** $\overline{RMSE}(^\circ)$			
**Angle**	**In-Sample**	**Out-Sample**	**All Samples**
Ankle	5.137 ± 1.863	4.761 ± 1.617	4.949 ± 1.744
Knee	8.195 ± 1.798	8.439 ± 1.843	8.317 ± 1.821
Hip	4.615 ± 1.013	4.638 ± 0.993	4.626 ± 1.003

Supplementary Table. 3. Ankle, knee and hip joint angle estimation $\overline{RMSE}(^\circ)$s with respect to In-Samples, Out-Samples and All Samples of WNN-GI and MLP-GI.

WNN-GI $\overline{\rho}$			
**Angle**	**In-Sample**	**Out-Sample**	**All Samples**
Ankle	0.945 ± 0.029	0.951 ± 0.026	0.948 ± 0.028
Knee	0.982 ± 0.014	0.980 ± 0.016	0.981 ± 0.015
Hip	0.990 ± 0.008	0.989 ± 0.009	0.990 ± 0.009
**MLP-GI $\overline{\rho}$**			
**Angle**	**In-Sample**	**Out-Sample**	**All Samples**
Ankle	0.846 ± 0.058	0.860 ± 0.052	0.853 ± 0.055
Knee	0.921 ± 0.026	0.918 ± 0.027	0.920 ± 0.027
Hip	0.937 ± 0.026	0.937 ± 0.027	0.937 ± 0.027

Supplementary Table. 4. Ankle, knee and hip joint angle estimation $\overline{\rho}$s with respect to In-Samples, Out-Samples and All Samples of WNN-GI and MLP-GI.

WNN-GI $\overline{NRMSE}(\%)$			
**Angle**	**In-Sample**	**Out-Sample**	**All Samples**
Ankle	10.005 ± 3.229	9.586 ± 3.136	9.796 ± 3.183
Knee	6.745 ± 2.230	6.804 ± 2.316	6.775 ± 2.273
Hip	5.438 ± 1.914	5.634 ± 2.105	5.536 ± 2.012
**MLP-GI $\overline{NRMSE}(\%)$**			
**Angle**	**In-Sample**	**Out-Sample**	**All Samples**
Ankle	17.153 ± 4.012	16.191 ± 3.618	16.672 ± 3.820
Knee	14.016 ± 2.525	14.159 ± 2.592	14.087 ± 2.559
Hip	12.589 ± 2.389	12.588 ± 2.287	12.588 ± 2.339

Supplementary Table. 5. Ankle, knee and hip joint angle estimation $\overline{NRMSE}(\%)$s with respect to In-Samples, Out-Samples and All Samples of WNN-GI and MLP-GI. In this study, $\overline{NRMSEs}$ were calculated to compare WNN-GI performance with $\overline{NRMSEs}$ reported by other literature.

WNN-GI In-Sample $\overline{RMSE}(^\circ)$			
Angle	**Sequence 1**	Sequence 2	Sequence 3
Ankle	2.947 ± 1.124	2.880 ± 1.129	2.900 ± 1.148
Knee	3.922 ± 1.326	4.024 ± 1.369	3.967 ± 1.343
Hip	2.029 ± 0.894	2.076 ± 0.875	2.102 ± 0.873
**WNN-GI Out-Sample $\overline{RMSE}(^\circ)$**			
Angle	**Sequence 1**	Sequence 2	Sequence 3
Ankle	2.772 ± 1.070	2.868 ± 1.248	2.868 ± 1.125
Knee	4.035 ± 1.411	3.856 ± 1.267	3.940 ± 1.384
Hip	2.114 ± 0.954	2.147 ± 1.003	2.191 ± 1.058
**WNN-GI In-Sample $\overline{\rho}$**			
Angle	**Sequence 1**	Sequence 2	Sequence 3
Ankle	0.945 ± 0.029	0.949 ± 0.027	0.947 ± 0.027
Knee	0.982 ± 0.014	0.981 ± 0.015	0.980 ± 0.015
Hip	0.990 ± 0.008	0.989 ± 0.008	0.989 ± 0.008
**WNN-GI Out-Sample $\overline{\rho}$**			
Angle	**Sequence 1**	Sequence 2	Sequence 3
Ankle	0.951 ± 0.026	0.946 ± 0.032	0.949 ± 0.031
Knee	0.980 ± 0.016	0.982 ± 0.014	0.981 ± 0.014
Hip	0.989 ± 0.009	0.989 ± 0.008	0.990 ± 0.008

Supplementary Table. 6. Ankle, knee and hip joint angle estimation accuracies of WNN-GI. Sequence 1 produced the best estimation accuracies (indicated in bold). Therefore, Sequence 1 accuracies are considered when calculating the summaries in Table.2-5.

MLP-GI In-Sample $\overline{RMSE}(^\circ)$			
Angle	**Sequence 1**	Sequence 2	Sequence 3
Ankle	5.137 ± 1.863	5.406 ± 1.830	4.801 ± 1.817
Knee	8.195 ± 1.798	9.575 ± 1.837	8.680 ± 1.856
Hip	4.615 ± 1.013	4.486 ± 0.835	4.094 ± 0.813
**MLP-GI Out-Sample $\overline{RMSE}(^\circ)$**			
Angle	**Sequence 1**	Sequence 2	Sequence 3
Ankle	4.761 ± 1.617	5.300 ± 1.662	4.856 ± 1.689
Knee	8.439 ± 1.843	9.344 ± 1.866	8.748 ± 1.735
Hip	4.638 ± 0.993	4.430 ± 0.918	4.150 ± 0.967
**MLP-GI In-Sample $\overline{\rho}$**			
Angle	**Sequence 1**	Sequence 2	Sequence 3
Ankle	0.846 ± 0.058	0.830 ± 0.058	0.842 ± 0.068
Knee	0.921 ± 0.026	0.898 ± 0.032	0.902 ± 0.039
Hip	0.937 ± 0.026	0.945 ± 0.018	0.954 ± 0.015
**MLP-GI Out-Sample $\overline{\rho}$**			
Angle	**Sequence 1**	Sequence 2	Sequence 3
Ankle	0.860 ± 0.052	0.832 ± 0.058	0.843 ± 0.066
Knee	0.918 ± 0.027	0.900 ± 0.033	0.903 ± 0.037
Hip	0.937 ± 0.027	0.945 ± 0.017	0.953 ± 0.015

Supplementary Table. 7. Ankle, knee and hip joint angle estimation accuracies of MLP-GI. Sequence 1 produced the best estimation accuracies (indicated in bold). Therefore, Sequence 1 accuracies are considered when calculating the summaries in Table.2-5.
